# Death from Ether in a Dental Office

**Published:** 1874-01

**Authors:** 


### SELECTED ARTICLES.

---

### ARTICLE II.

### *Death from Ether in a Dental Office.*

From the *Boston Medical and Surgical Journal* we learn the following particulars concerning a case which has caused considerable excitement in Boston :

The announcement in the morning papers of November 11th that on the previous day the death of a lady by ether had occurred in the practice of Dr. Eastham, a dentist in this city, caused much excitement in professional circles. The death had taken place about noon, but very few, except those particularly interested, were aware of it till the next day. The coroner, Dr. Ainsworth, who was called in directly after the accident, formed a jury of physicians and apothecaries and ordered an autopsy. This was made the next morning by Dr. R. H. Fitz, Pathologist to the Massachusetts General Hospital, and on the same day the jury met, and, having viewed the body, ajourned till the 14th. Before our last number went to press, we had barely time to collect sufficient evidence to justify the statement which we made, that the anæsthetic was either chloroform or a mixture of chloroform and ether. The latter proves to be the one used. The jury met again on the 14th, and having heard a part of the evidence re-adjourned till the evening of Wednesday the 19th. We shall refrain from comment on the evidence till the verdict is rendered, but present the following account of the proceedings which we condense from a complete stenographic report taken especially for us. On Nov. 14th, the first witness was Dr. Edson, who testified that he had twice attended Mrs. Crie, the deceased, during her confinements, but had never given her an anæsthetic, though she desired it. This was owing to his disapproval of anæsthetic during labor, except in rare cases. He would have given one to the deceased as readily as to any patient in her case.

Dr. Fitz was next called, and read the following account of the autopsy :—

Examination made twenty-one hours after death. Body preserved in ice; rigidity well marked; no discoloration of face or anterior portions of the body; skull cap and dura mater normal; longitudinal veins empty; moderate amount of blood in the veins of the arachnoid; nothing abnormal observed at the base of the brain. The blood-vessels in this region contained but little blood; cerebral substance firm, containing much less blood than usual, not particularly moist; absence of any anatomical changes; ventricles apparently normal. Pericardium healthy. Heart moderately contracted, unusually small and of usual color; aorta of less than the normal calibre, walls unusually thin and elastic; cavities of the heart contained dark fluid blood, of no unusual odor or color: right side of the heart contained more blood than the left; valves healthy, muscular substance apparently normal. Pleural cavities healthy, containing a small amount of reddish fluid. Lungs of a bluish-red color, the posterior dependent portions quite dark; tissue contained air and a somewht increased amount of blood; absence of any special degree of œdema; in upper lobes of both lungs a rare, small, cheesy nodule. The larynx, trachea, bronchial tubes and the larger vessels at the root of the lungs free from changes. Spleen of normal size and firmness, the color dark blue. Kidneys unusually firm, capsule rather more adherent than usual; in sections, the organ was of a grayish slate color; bloodvessels including the malpighiam organs, unusually distinct from the presence of blood; tubular structure apparently healthy. Bladder healthy. Uterus and ovaries well developed; an old *corpus luteum* present; the lining membrane of the body of the uterus unusually injected, covered with a viscid, bloody fluid. Liver of normal size, dark color, containing rather more blood than usual, otherwise healthy; stomach and intestines presented no unusual appearances.

The anatomical examination gave no evidence of recent disease of any of the organs, or of chronic alterations sufficient to account for death; the fluid conditions of the blood, the diminished amount in the brain, and the increased amount in the thoracic and abdominal organs were abnormal, and might have been the result of various causes; the diminished size of the heart and of the aorta were probably of congenital origin.

*Question.* Do you consider the absence of blood in the brain and cerebral cavities as abnormal? *Answer.* Yes, sir.

*Q.* Do you ever find the blood liquid so long after death, except where chloroform is used? *A.* Yes, sir; it is so in any case of death from asphyxia, in cases of poison from certain gases, and in cases of some very malignant forms of disease where decomposition is very rapid.

*Q.* I suppose a perfectly healthy woman would not be likely to have this sudden change take place in her without some cause similar to those you have mentioned? *A.* Very unlikely.

Dr. Eastham then testified that he graduated in medicine in 1841, had practised dentistry nearly all the time since, and had used anæsthetics from their introduction. The deceased had been his patient for twelve or fourteen years, during which he had on several occasions given her anæsthetics, chloroform, ether and gas, both severally and in combination. The deceased came to his office in the forenoon of the 10th, and there met Mrs. Sawyer, whose tooth he extracted after giving nitrous oxide. Mrs. Sawyer urged the deceased to take gas, but she insisted upon ether. He made of a little mixture *chloroform and ether.*

*Question.* You made a mixture at the time? *Answer.*—Yes, sir; I usually do that way.

*Q.* Please tell whether or not on this occasion you measured the quantity? *A.* No. I have been so familiar with it that I usually guess at the proportion. I never measure it. I always calculate to have more ether than chloroform.

*Q.* How much of this mixture did you make? *A.* Not more than an ounce or an ounce and a half.

*Q.* How did you administer it? *A.* I always administer it on a sponge. I always drop the window at the top, so as to have fresh air. I pour on to this sponge (it is a hollow one about the bigness of my two hands) about a big teaspoonful, as near as I can judge.

*Q.* That would have been about a third of the mixture? *A.* No, not so much as that. I always begin gradually in applying it, first holding the sponge a little distance from the nose and then moving it nearer. As she began to breathe it, she said, "Give me enough this time, sure." This she repeated three times. I did not fully etherize her, nor did I intend to. After she had breathed two or three minutes, I said to her, "I am going to take this tooth out." She shook her head, as much as to say she was not ready but I took hold of the tooth. She straightened back, groaned and screamed a little as if in pain. After I had pulled the tooth, she went back into a sort of hysterics, and became rigid, as if in spasms.

*Q.* At this point in the case, did you notice her lips, whether they were pale? *A.* Not much.

*Q.* Any change in her countenance? *A.* Not much.

*Q.* Did you notice her eyes? *A.* They were set wide open, like one in a spasm.

*Q.* You did not notice whether there was anything particular about the lips? *A.* No.

*Q.* Did you try the pulse at that time? *A.* No. I seized a napkin, moistened with water, and gave her a splash on the forehead. She seemed to revive, and I saw a flush of color come over her face. I set her up and took my ammonia water and applied that to her nose; then I spoke to Mrs. Sawyer. Mrs. Crie was sitting up in the chair, inclined a little forward at that time, and I was applying ammonia and water to the face. Mrs. Sawyer came in, and I asked her to loosen her dress, which she did. Then I saw a change again, back to paleness, and I said, "Call the other doctors." Dr. Osgood arrived first. We unloosed Mrs. Crie's corsets. Dr. Osgood rubbed her spine, and I sent the porter after another physician. We continued to rub her and apply very strong ammonia, and, finally, after Dr. Damson came in, we removed her to the large room and, raising her arms, tried in every way to set up a respiration. We sent for a battery and used that. We worked over her till we all came to the conclusion that she was past all restoration.

*Q.* Can you tell us how long after she fell back into this spasm it was before respiration ceased? *A.* I should say about fifteen minutes.

*Q.* How long did the the flush continue? *A.* It might have been two minutes.

*Q.* Then, as I understand, she fell back at once? *A.* As soon as the shade went back, I called for help. After administering these anæsthetics, there are two peculiar shades. There is the shade for faintness, and a shade from sickness at the stomach, and they are perfectly distinct.

*Q.* What was your opinion of this peculiar shade then? *A.* I thought it was a pallor from faintness.

*Q.* From the time she had this spasm and during the time you were administering the ammonia, was she sitting up in the chair? *A.* Yes, sir; but after the doctors came in they removed her to the waiting-room and laid her down.

*Q.* Was she breathing then? *A.* She was dead.

*Q.* How long had you begun the administration of ether before you extracted the tooth? *A.* About a minute or a minute and a-half.

*Q.* During that time did you feel no pulse? *A.* Never do that. Always watch the side of the head, the temporal artery.

*Q.* Do you think there is any danger of death occurring from giving ether alone? *A.* I never had anything that appeared like it myself; nor in chloroform.

*Q.* You have not considered then that there was any danger? *A.* No, I do not—that is, unless you administer it as they do in England, I should think they would kill every other one, by using a napkin as they do. But if chloroform be given as I give it on a sponge, with plenty of fresh air, I don't consider it any more dangerous than ether; but a person must discriminate between individuals, whether he would give ether, or gas, or chloroform or anything, and these things must be learned by practice.

*Q.* You considered her to be a person lacking somewhat in vitality, and therefore you didn't choose to put her fully under the influence of it (the anæsthetic?) *A.* Yes, sir.

*Q.* Do you consider either of these anæsthetics more dangerous than the others? *A.* I suppose chloroform would decompose blood quicker than ether.

*Q.* Do you know of any difference in chloroform? *A.* I have never used but one kind, Squibb's.

*Q.* In what way do you keep it? *A.* Always in a dark closet and corked as tight as I can.

*Q.* Do you know of any difference in the quality of ether? *A.* No, only from the seller's opinion of it. I use Powers and Weightman's concentrated.

*Q.* How much of this mixture did you generally make at a time? *A* Not more than a couple of ounces at once.

*Q.* What was the proportion of chloroform that you generally intended to have in? *A.* Less than half, by volume.

*Q.* Did you keep that mixture a long time? *A.* No, but I would most always add more ether if it had been standing a little while.

*Q.* Did you state that you made this mixture you administered to Mrs. Crie that day? *A.* I had a little in a bottle and I added more to it, before I gave it to her. I had used it a week before.

*Q.* What is your reason for adding in chloroform to the ether? *A.* Well, I think it is safer. Ether is a great stimulant, and when you have a little chloroform, the patients are not so noisy or excited as they are under pure ether. That is my reason, not that I feared one or the other.

*Q.* You would not hesitate to give any quantity of chloroform? *A.* No. sir. If amputation was to be performed I had as soon use chloroform as ether.

*Q.* On the whole, which anæsthetic do you consider the most safe? *A.* I think I should use ether for safety. Ether and chloroform combined, in my idea, is much better than either of them alone.

*Q.* Do you feel any anxiety when about to administer chloroform or ether or the mixture? *A.* No.

We understand that at the next meeting, Dr. Wood will give the results of his analysis of the anæsthetic, that Mrs. Sawyer will be examined, and that expert testimony on the use of anæsthetics will be heard.

We have said that we should make no comments on the evidence while the investigation is in progress, but we may without indiscretion express our gratification at Dr. Ainsworth's course in giving the affair a thorough and public examination. This should be done in every case of death from anæsthesia.

---

The coroner's jury in the case of Mrs. Crie met on Nov. 19th and heard the remainder of the evidence. Mrs. Lee, a friend of the deceased, testified that, two days before her death, the latter appeared in excellent health, that she had never known her to complain of trouble in her heart, that she dressed loosely, and was not then nursing her child. Mrs. Sawyer was then called. She repeated very closely Dr. Eastham's account of the events preceding the inhalation, and of such efforts at resuscitation as she witnessed. She thought the deceased was laced very tightyl. She advised her to take gas, because she had herself recovered from it very nicely, and previously had taken ether and felt it for two weeks.

*Question.* Are you sure it was ether? *Answer.* It was ether and chloroform.

*Q.* Did Mrs. Crie insist on taking ether, or did she say something about chloroform? *A.* She said ether.

Dr. G. H. B. Flagg is a dentist in Boston. He was in Dr. Eastham's room while Mrs. Crie was dying. He felt her pulse; it was very slow, not more than twenty-five, and feeble, and to him it was apparent that she could not live.

*Q.* Have you been in the habit of giving chloroform yourself? *A.* No, sir.

*Q.* Either purely or combined with ether? *A.* Four times in the last ten years I have given a mixture of chloroform and ether.

Dr. Flagg said that he preferred not to give chloroform, but did not know enough about it to say it was dangerous.

*Q.* Were you in the habit of giving gas to Dr. Eastham's patients? *A.* I usually assisted him.

*Q.* And he has given ether to your patients? *A.* Four times, sir, during the last ten years.

*Q.* Then when you stated you had administered chloroform, you meant it had been administered by Dr. Eastham? *A.* Yes, sir.

*Q.* You knew it was the mixture of chloroform and ether? *A.* Yes, sir.

Dr. H. D. Osgood.—Q. You are a practising dentist? A. Yes, sir.

Q. You were called into Dr. Eastham's office between eleven and twelve o'clock on Monday, Nov. 10th? A. Yes, sir.

Q. Will you state to the jury what you saw there? A. Mrs. Sawyer came into my room at that time and said that Dr. Eastham wanted to see me, for a lady who had taken ether had fainted.

Q. Did she mean ether, or chloroform, or a mixture? A. I don't know; she said ether. I went into his office, and saw a lady in the operating chair.

Q. What was her position? She was inclined forwards. She seemed very low. I examined her pulse, and could not detect any at all.

Q. Did you detect any respiration? A. No, sir, I did not. Examining her clothing, I found she had on corsets, and that they were quite tightly laced. We applied ammonia and water, and Dr. Eastham slapped her face vigorously with a towel.

Q. Did she at all revive in any way? A. Not to my knowledge.

Q. You could not feel any pulse or detect respiration. Do you think she was dead? A. It was my opinion that she was dead.

Q. That was when you first went in, soon after Mrs. Sawyer called you? A. Yes, sir.

Q. Dr. Osgood, you have been for a long time practising dentistry? A. Yes, sir.

Q. You have been in the habit of giving ether? A. Yes, sir.

Q. Have you been in the habit of giving ether and chloroform? A. Yes, sir.

Q. Do you consider, from the experiences you have had in the use of it, that chloroform is safe, either alone or combined with ether or alcohol? A. I consider it so, or I should not have used it.

Q. Do you give it alone? A. Yes, sir, I have done so many times.

Q. Have you given the mixture? A. Yes, sir.

Q. In what proportions? A. One-third chloroform and two-thirds ether.

Q. Has Dr. Eastham given it for you? A. Yes, sir.

Q. You always knew it was ether and chloroform? A. Yes, sir.

Q. Did the patient know it? A. I could not say.

Q. What did they call for? A. I don't know, sir.

Q. They must have required an anæsthetic or you would not have given it? A. True.

Q. Did they call it an anæsthetic? A. No, sir.

Q. What did they ask for? A. I cannot tell, sir. I think quite likely they called for ether.

Q. Did they ever call for chloroform? A. Yes, sir, very often.

Q. When they called for chloroform, did you give them the mixture? A. I may not have given them either, but gas instead. We never give ether or chloroform when we can get them to inhale gas instead. Sometimes, one demands either ether or chloroform, and then we give it to him.

Q. You give the mixture when they call for the ether or chloroform! A. Yes, sir.

Q. Have you ever seen any ill effects from ether and chloroform mixed? A. No, sir.

Q. The uniform strength has been about one-third chloroform? A. Yes, sir.

Q. By weight? A. No, sir, by bulk. I never considered it a very great matter whether one third, a little more or a little less.

Q. Do you give anæsthetics now as much as you did ten years ago? A. Do you mean ether, and chloroform and nitrous oxide?

Q. Yes. A. I do a great deal more.

Q. Is the use of ether and chloroform on the increase or decrease with you? A. I don't give as much as I formerly did.

Dr. E. S. Wood, acting professor of chemistry at the Harvard Medical School, gave the following account of his analysis:

I received a small glass-stoppered vial containing liquid; a portion of a liver, a spleen and kidney, and the contents of a stomach. The vial contained 1·39 ounces. The odor of the liquid resembled that of ether mixed with chloroform, the odor of chloroform being strongly perceptible. The specific gravity of the fluid=1·043, which corresponds to that of a mixture of six parts by bulk of ether with four of chloroform, if allowance be made for an increase in the density of the two when mixed. A mixture of sixty per cent, of ether with forty of chloroform had a specific gravity of exactly 1·043 at 68 degrees Fahr., and had lost about 1-100 of its volume. A mixture of sixty parts ether with forty chloroform will not occupy one hundred parts by volume, but only 98·945 parts, and its specific gravity, instead of being 1·032, as if no condensation took place, will be 1·043. The mixture contained no hydrochloric or acetic acids and no chlorine, showing that both the ether and chloroform were free from any deleterious impurity, a small amount of alcohol only existing as an impurity. The liquid answered the tests both for chloroform and ether. By bulk, it was sixty per cent ether, and forty per cent. chloroform, and by weight 58·14 per cent. ether and 41·86 per cent. chloroform. The blood had no odor, either of chloroform or ether, and neither of these liquids was detected by analysis; and the same is true of the orgaus which were carefully analyzed.

Q. You are somewhat familiar with statistics of anæsthetics, are you not? A. I have seen some statistics.

Q. Have you it in your own power to tell the jury the statistics relative to the mortality occasioned by the use of ether or chloroform, or a mixture of the two? A. The only statistics which I have seen were some which were published in Chicago in 1870, and these were reprinted, or rather copied into the last annual report on the practice of pharmacy and toxicology.

Q. Will you please state what these were? A. Roughly the proportion of deaths to cases in ether was one in twenty-five thousand; to cases in chloroform, one in twenty-five hundred; to cases of a mixture of chloroform and ether, about one in five thousand.

Q. If you had been handed all the articles, without the chloroform and ether, could you have given any opinion as to the cause of the person's death? A. No, sir.

Q. Did I understand you that there were no odors in the blood? A. Yes, sir. The blood was strongly alkaline.

Q. What do you think is the smallest amount of chloroform that would cause death? A. The smallest reported, as I remember, was from fifteen to twenty drops by inhalation, one drachm taken by the mouth into the stomach, and one drachm of a mixture containing one part chloroform to four of ether by bulk, that is, one teaspoonful.

Q. You mean that dose has caused death? A. Yes, sir, immediately; that is within a few minutes.

Q. Is there any record of the presence of any poison in the blood of any of these cases reported? A. It has sometimes, but rarely, been possible to detect chloroform in the blood. The analysis after death from ether, in case of animals, have been unsatisfactory, and in case of death from chloroform it is only sometimes possible to detect it.

Q. Have you any idea of the cause of absence of congulation in the blood? A. No, sir The spectroscopic examination of the blood gave a normal appearance.

Dr. Henry J. Bigelow—Q. You have heard the testimony in this case; you have it under oath that this lady had breathed from two to four drachms of a mixture of ether and chloroform such as you have heard stated: now what is your opinion as to the cause of death? A. She died of breathing chloroform; there is no question about it.

Q. You have no doubt that the chloroform which was used in that mixture was the cause of death? A. It was the cause of death.

Q. She took about two-fifths chloroform and three-fifths ether according to bulk; would that amount of ether be sufficient to cause death? A. It would not possibly cause death.

Q. Would it be safe for a child six years old? A. I cannot conceive that it would effect it deleteriously.

Q. Two-fifths chloroform? A. Might kill an adult.

Q. Have you ever in your experience known of any death by chloroform? A. I have been present at but one.

You are familiar with the literature on that subject. From your reading, how many cases are you prepared to answer for? A. I am wholly unable to give a number. They are numbered by hundreds, and it is proved that many are not reported.

Q. Have you ever known of a case of death from ether properly administered? A. No, sir.

Q. Do you, from your own knowledge or by reading, believe there ever was a case of death from ether properly administered? A. There is a fallacy in the proposition put in that way. Ether is a powerful agent, and if a man is feeble or dying, it would contribute to his death like a dose of opium or anything else that has weight and force and power in it. But that is not the real question as between ether and chloroform, for both of them are powerful agents. The real question is, has chloroform, besides this narcotic power, some very poisonous influence which acts upon the system and in which it differs from ether; has chloroform such a power, has ether, or have both? I answer, chloroform has and ether has not; chloroform kills suddenly and ether cannot.

Dr. S. Cabot.—Q. As a result of your experiences, do you think it dangerous in any way to give ether by inhalation? A. I don't, sir, with, of course, proper precautions.

Q. From your knowledge and personal experience, do you consider it safe to give chloroform? A. No, sir, I do not.

Q. Even properly; with all precautions possible to be taken? A. I don't think it safe.

Q. Judging from your knowledge, what do you think caused the death of Mrs. Crie? A. Inhalation of chloroform.

Q. Do you think that two-fifths of a tablespoonful of chloroform, as taken by bulk, would be sufficient to produce that effect? A. I do, sir.

Q. In your judgment, can a person in ordinary good health take ether to produce death, say in the course of ten or fifteen minutes? A. No, sir.

Drs. Henry G. Clark, George H. Gay and R. M. Hodges testified to the same effect. On the next evening, the jury met again, and presented the following verdict:—

That Mary F. Crie came to her death on Monday, the 10th day of November, 1873, between eleven A. M. and one P. M., in the office of Dr. Charles Eastham, a dentist, No. 25 Tremont street, Boston, and that her death was caused by the inhalation of chloroform administered in a mixture of chloroform and ether by the said Dr. Eastham.

The jury use this opportunity to caution the public against the inhalation of so dangerous an agent as chloroform for the production of insensibility to pain. In the opinion of the jury the inhalation of sulphuric ether is safe, while the inhalation of chloroform, either alone or mixed, is always attended with danger.

It was signed by Ezra Palmer, M. D., John A. Lamson, M. D., Geo. Fabyan, M. D., George Lotz, M. D., Thomas Restieaux and Thomas Doliver.

This case has attracted much attention, not only from the attempt made just after the accident to pass the death off as one from ether, but also, when it became evident that it was due to chloroform, from anxiety to see what would be the conclusions of a Boston jury. The verdict is all that could be desired, as it expresses emphatically the feeling of the profession, and we do not find fault that Dr. Eastham was spared the well-deserved censure which he must have expected. The misfortunes of the past should be remembered only as warnings for the future. The use of chloroform is least justifiable where ether is best known; there is less excuse for its use in America than in Europe, and least of all in this city. After this verdict, nothing but very exceptional circumstances will warrant its administration. It appears in the evidence that several dentists are in the habit of giving whichever anæsthetic they see fit, regardless of the request of the patient. We hope that this custom is not general, and would advise any who may persist in it not to be too sure that after another patient, who shall have asked for ether, has been killed by chloroform, the verdict may not contain, besides other disagreeable words, the adjective "criminal."

---

